# Nursing the Sick

**Published:** 1872-12

**Authors:** 


### NURSING THE SICK.

A FRAIL young lady of fortune and family left England for the seat of war in the Crimea, impressed with the idea that many a brave soldier died for want of proper care. On her arrival, she inaugurated a system of nursing which, in a few months, diminished the mortality from sickness, nearly one half. The regard of those in the hospitals where she paid regular visits was such as to amount almost to an adoration. When the poor sufferers could not get to her, they would place themselves in a position where her shadow would pass over them, and the name of

FLORENCE NIGHTINGALE

will pass down to the ages as the representative of one of the most beautiful characters in history.

In view of the fact that an incalculable amount of human suffering and premature death can be prevented by judicious nursing, and that in large towns and cities there is always an urgent demand, at high wages, for intelligent, competent, and experienced attendants in the sick chamber, the suggestion is made that a new and lucrative avenue to competence might be opened to women who would qualify themselves as nurses for the sick.

